# Overexpression of *ß-Ketoacyl Co-A Synthase1* Gene Improves Tolerance of Drought Susceptible Groundnut (*Arachis hypogaea* L.) Cultivar K-6 by Increased Leaf Epicuticular Wax Accumulation

**DOI:** 10.3389/fpls.2018.01869

**Published:** 2019-01-11

**Authors:** Uppala Lokesh, Boya Venkatesh, Kurnool Kiranmai, Ambekar Nareshkumar, Vennapusa Amarnathareddy, Gunupuru Lokanadha Rao, Anthony Masilamani Anthony Johnson, Merum Pandurangaiah, Chinta Sudhakar

**Affiliations:** ^1^Plant Molecular Biology Laboratory, Department of Botany, Sri Krishnadevaraya University, Anantapur, India; ^2^Department of Plant, Food, and Environmental Sciences, Dalhousie University, Truro, NS, Canada

**Keywords:** groundnut, drought stress, *AhKCS1*, epicuticular wax, non-stomatal water loss, transgenic plants

## Abstract

Drought is one of the major environmental constraints affecting the crop productivity worldwide. One of the agricultural challenges today is to develop plants with minimized water utilization and reduced water loss in adverse environmental conditions. Epicuticular waxes play a major role in minimizing water loss. Epicuticular wax covers aerial plant parts and also prevents non-stomatal water loss by forming the outermost barrier from the surfaces. Epicuticular wax content (EWC) variation was found to be affiliated with drought tolerance of groundnut cultivars. In the current study, a fatty acid elongase gene, *KCS1*, which catalyzes a rate limiting step in the epicuticular wax biosynthesis was isolated from drought tolerant cultivar K-9 and overexpressed in drought sensitive groundnut cultivar (K-6) under the control of CaMV35S constitutive promoter. Transgenic groundnut plants overexpressing *AhKCS1* exhibited normal growth and displaying greenish dark shiny appearance. Environmental scanning electron microscopy (ESEM) revealed the excess of epicuticular wax crystal depositions on the transgenic plant leaves compared to non-transgenic wild type plants. The findings were further supported by gas chromotography–mass spectroscopic analysis (GC-MS) that revealed enhanced levels of fatty acids, secondary alcohols, primary alcohols, aldehydes, alkanes, and ketones in transgenics compared to wild types. The *AhKCS1* overexpressing transgenic groundnut plants exhibited increase in the cuticular wax content, reduction of water loss, lower membrane damage, decreased MDA content, and high proline content compared to that of non-transgenic groundnut plants. Our findings suggest that the *AhKCS1* gene plays a major role in combating drought stress by preventing non-stomatal water loss in drought sensitive groundnut cultivar (K-6).

## Introduction

The major limiting abiotic stress factors like drought, salinity, and temperature, affect the growth, development of crop plants, and finally result in severe crop yield losses (Joshi and Karan, 2013; Fang and Xiong, 2015; Wang et al., 2016). To cope up with these inimical environmental conditions, plants developed adaptive mechanisms through integrated physiological, biochemical and molecular responses. Tolerance to drought is a complicated event associated with many adaptive traits through which plants would efficiently utilizes moisture under water deprived conditions. Deep root system, leaf morphology, cuticular wax depositions, and cutinization of the leaf surfaces are important physical adaptations that provide protection to plants against drought (Samuels et al., 2008; Lee and Suh, 2015). Cuticle, an extracellular lipophilic layer containing characteristic chemical composition such as waxes, is the primary defense mechanism that controls water loss (non-stomatal) from the surface organs of the plants into the encompassing environment (Jenks et al., 2002; Mao et al., 2012). The epicuticular wax accumulation on the aerial surfaces is an important adaptation in the course of drought stress seen in several crop plants such as sorghum, peanut, and pea (Sanchez et al., 2001; Samdur et al., 2003; Burow et al., 2008).

Disparity in the cuticular wax amount, composition and morphology of crystals was observed at different developmental stages, between plant species, between organs of the same plant and in counter to environmental conditions (Schreiber and Riederer, 1996; Barthlott et al., 1998; Riederer and Schreiber, 2001; Jetter et al., 2007; Samuels et al., 2008; Joubes et al., 2008; Mamrutha et al., 2010; Buschhaus and Jetter, 2011; Bernard and Joubes, 2013; Lee and Suh, 2015). In many plants like Arabidopsis (Yang et al., 2011), rice (Islam et al., 2009), sesame (Kim et al., 2007), wheat (Adamski et al., 2013; Zhang et al., 2013, 2015; Wang J. et al., 2014), cotton (Bondada et al., 1996), tree tobacco (*Nicotiana glauca*) (Cameron et al., 2006), and alfalfa (Shephered and Griffiths, 2006; Kosma and Jenks, 2007; Kosma et al., 2009), drought triggered the accumulation of epicuticular waxes. Waxes are the complicated amalgam of the very-long-chain fatty acids and its derivatives, synthesized by the elongation of C16-C18 to C32-C40 in endoplasmic reticulum, through a series of Fatty Acid Elongase enzymes (FAE) such as ß-Ketoacyl Co-A synthase (KCS), ß-Ketoacyl Co-A Reductase (KCR), Hydroxyacyl Co-A Dehydratse (HCD), and Eonyl Co-A Reductase (ECR), gets transported on to the exterior of leaves, stem, and deposited as crystals (Samuels et al., 2008; Kunst and Samuels, 2009; Haslam and Kunst, 2013; Lee and Suh, 2015). The wax ingredients, such as the secondary alcohols, aldehydes, alkanes, and ketones, are derived from the alkane-forming pathway and primary alcohols and wax esters, are synthesized by the alcohol-forming pathway (Bernard and Joubes, 2013).

The fatty acid elongation is the rate-limiting step in cuticular wax biosynthesis, initiated by *KCS* gene that regulates the length of the chain and its substrate specificity (Millar and Kunst, 1997; Denic and Weissman, 2007). At the molecular level, several wax biosynthesis genes were described in crop plants such as *Arabidopsis* (Todd et al., 1999; Chen et al., 2003; Go et al., 2014), rice (Islam et al., 2009; Qin et al., 2011; Mao et al., 2012; Zhou et al., 2013, 2014), oat cultivars (Bengtson et al., 1978), barley (Richardson et al., 2007), *Brassica napus* (Pu et al., 2013), and *Brassica rapa* (Zhang et al., 2014), banana (Sampangi-Ramaiah et al., 2017). Genes encoding the *KCS* (ß-ketoacyl-CoA synthase) were identified from the *Arabidopsis thaliana* (James et al., 1995) are subjected to VLCFA synthesis such as *KCS1* (Todd et al., 1999), *CER6* (Fiebig et al., 2000; Hooker et al., 2002), *CER1* and *CER3* (Rowland et al., 2007; Bernard et al., 2012), and in the generation of cuticle such as FATB (Bonaventure et al., 2003) and *FDH* (Pruitt et al., 2000). In crop plants, the biosynthesis of epicuticular waxes was activated by the *KCS* gene (Jung et al., 2014). In apple (*Malus domestica* Borkh.) overexpression of *KCS1* and *KCS4* resulted in the build-up of epicuticular wax (Albert et al., 2011).

Groundnut (*Arachis hypogaea* L.) is ranked the sixth most significant oilseed crop in the world, cultivated in 25.54 million hectares world over having a total production of 42.87 million tons globally (Foreign Agricultural Service United States Department of Agriculture [FAS/USDA], 2017). As Groundnut is mainly a rain-fed crop, its productivity is deprived by moisture stress in semi-arid tropics. Inspite of its wider adaptability under intermittent moisture stress conditions, the realized yields are substantially low (Kumar and Kirti, 2015). Groundnut has relatively lesser root to shoot ratios, thence low water mining abilities. The EWC of the crop minimizes the water loss thereby improve water conservation strategies under drought stress. Therefore, in the current study, an attempt was made to engineer the groundnut plant to combat the severe water losses by enhancing the epiculticular wax production. The wax content on the leaf surface of groundnut cultivars JL24, K9 (Solanki and Sarangi, 2015), Harithandra, Vemana, Anantha, K5, K6, and K9 (Lokesh et al., unpublished data) are reported. To date there has been no successful attempt to enhance the EWC through generation of transgenics by altering the wax biosynthesis pathways. Hence, we isolated a *KCS1* gene from a drought tolerant cultivar K-9 and overexpressed in drought susceptible cultivar K-6 to enhance stress tolerance.

## Materials and Methods

### Cloning of Full Length Gene *KCS1*

Total RNA from groundnut cultivar K-9 was isolated from tender leaves using Trizol method. The quality of RNA was checked and the cDNA was synthesized using oligodT primer and Revert aid Reverse transcriptase enzyme (Thermo Scientific, United States) as per manufacturer’s instructions. *KCS1* primers were designed using Primer3 tool, and the primers were synthesized at Eurofins Genomic, Bangalore, India. Then PCR was performed on a Gradient Thermal Cycler (Eppendorf, Germany) using *KCS1* specific forward (5′-ATGCCTCCCATGTTGCCGGA-3′) and reverse primers (5′-CTAGAGCTTAACGATCTCAGG-3′) using Takara Ex Taq DNA polymerase. The reaction mix comprises 1× Taq buffer, 0.2 mM dNTPs, 3 pmol/μl of forward and reverse primers, 20 ng of cDNA with thermal cycling conditions of initial denaturation at 94°C for 4 min followed by 35 cycles of denaturation at 94°C for 1 min, annealing at 62°C for 1 min and extension at 72°C for 1 min and a final extension at 72°C for 10 min. The amplified PCR product was gel purified using GeneJet gel extraction kit (Thermo Scientific, Germany) and cloned into T/A (pTZ57R/T) cloning vector (Thermo Scientific, United States) as per manufacturer’s instructions. PCR positive clones were sequenced commercially at Eurofin Genomics, Bangalore, India. Contig assembly was done using Bioedit Software. The sequence was submitted to Genbank database. Phylogenetic tree for *KCS1* gene sequences from various plant species was constructed using Mega software v. 6. Bootstrapped rooted Neighbor Joining tree was constructed with 1000 bootstrap replicates and 111 random odd number, with KCS2 gene sequence from *Arabidopsis thaliana* as an out group taxa.

### Construction of *AhKCS1* Overexpression Vector

The full-length *AhKCS1* gene was PCR amplified from using Plasmid DNA as template from the sequence confirmed clone using Forward primer 5′-*CTCGAG*ATGCCTCCCATGTTGCCGGA-3′ and Reverse primer 5′-*GGTACC*CTAGAGCTTAACGATCTCAGG-3′ with standard conditions of PCR as described above. The amplicon was cloned into pTZ57R/T using InsTA clone^TM^ PCR Cloning Kit (Thermo Scientific). The *KCS1* gene was then released by *Xho*I and *Kpn*I restriction enzymes and sub-cloned into intermediate vector pRT101. The CaMV 35S:*AhKCS1*: Tnos cassette was released by digestion with *Pst*I and then cloned into binary vector pCAMBIA2301. The recombinant overexpression construct designated as pC2301-*AhKCS1* (CaMV35S: *AhKCS1*:Tnos) was mobilized into *Agrobacterium* strain EHA105 by freeze thaw in liquid nitrogen with *NptII* as selectable marker (Sambrook et al., 1989). Positive agro-clones were further used for *in planta* transformation of groundnut cultivar K-6.

### Groundnut K-6 Cultivar *in planta* Transformation and Regeneration

Transformation in groundnut and genesis of the primary transformants, tissue culture-independent *in planta* transformation procedure was employed, as standardized and reported by Rohini and Rao (2001). The seeds of groundnut (*Arachis hypogaea* L.cv. K6) were sterilized with HgCl_2_ (0.1%, w/v) for 1 min and washed five to six times in sterile water. Seeds were imbibed overnight in sterile water and were placed on the petriplates lined with soaked filter paper in dark for 3–5 days. Using a sterile needle, freshly emerged plumules from the seedlings were pricked at the meristem and wounded. Then the wounded explants were immersed in the *Agrobacterium KCS1* culture for 2 h. After that the seedlings were disinfected with cefotaxime 100 mg/l, then rinsed in sterile water and transferred to soilrite (vermiculite equivalent). Plants were grown in soilrite in plant growth chamber (Model – Adaptis, Conviron, Canada) under controlled conditions of 25 ± 3°C for 15 days. Plants were watered upon requirement during the growth. Then the plantlets were transferred from soilrite into pots with soil and manure, acclimatized at room temperature. After 5 days, pots were transferred to green house. As expected to be chimeras, no attempt was made to characterize the plants in this generation. To select putative transformants in T1 generation, seeds were allowed to germinate on MS half strength media containing 200 mg/l kanamycin in tissue culture bottles for 10 days in the growth chamber. Germinated seedlings (putative transformants) were shifted to pots with soilrite and 2:1 (garden soil and farmyard manure) and allowed to grow under room temperature for 10 days, until transfer to green house with transgenic containment facility (Supplementary Figure 7).

### Molecular Analysis of Transgenics

#### RT-PCR

Genomic DNA was extracted from the leaf tissue of transgenic plants by CTAB method (Doyle and Doyle, 1990). To confirm the integration of transgene, PCR was performed using neomycin phosphotransferase II (*NptII*) forward (5′-TGAATGAACTGGAGGAG-3′) and reverse (5′-AGCCAACGTATGTCCTGAT-3′) primers and *GUS* forward (5′-TTCGCGTCGGCATCCGCTCAGTGGCA-3′) and reverse (5′-GCGGACGGGTATCCGGTTCGTTGG-3′) primers and *AhKCS1* gene-specific forward (5′-ATGCCTCCCATGTTGCCGGA-3′) and reverse primers (5′-CTAGAGCTTAACGATCTCAGG-3′).

#### SYBR Green-Based Real Time qRT-PCR Analysis

For evaluating transgene expression, Real time PCR was done from total RNA of transgenic plant leaf samples. To remove genomic DNA contamination, all the RNA samples were treated with DNase1 and then used to synthesize cDNA. Five micrograms of total RNA (template) was used with the RevertAid Reverse Transcriptase (Themo Scientific, United States) as per manufacturer’s protocol. To analyze the levels of transgene expression, *AhKCS1* gene-specific primers forward (5′-CATCTCCATAGACCTAGCACGC-3′) and reverse (5′-CCGCCCATCCTGAACAAG-3′) were used in SYBR green based Real time PCR analysis. The house keeping gene actin forward (5′-TCCATAATGAAGTGTGATGT-3′) and reverse (5′-GGACCTGACTCGTCATACTC-3′) were used as internal controls for all the real time reactions. Real time analysis was standardized using 1× Power SYBR green mix, 0.9 pmol of each forward and reverse primers in 48 well plates on Applied Biosystems Step One Real time PCR machine.

### Scanning Electron Microscopy Analysis of Leaf Surfaces

Scanning electron microscopy (SEM) was employed for study of surface wax deposition of the leaves collected from *AhKCS1* transgenic groundnut lines, mock, and wild type plants. At room temperature, the samples were air-dried in a desiccator and then dissected carefully. Three to five millimeter fully dried pieces were stuck to the double adhesive tape of the aluminum stubs, and the surfaces were envisaged using Environmental Scanning Electron Microscope (QUANTA 250 ESEM, IOP, Bhubaneswar, India).

### Estimation of Wax Composition by GC-MS Analysis

Wax from the leaf exterior was obtained from transgenic groundnut lines (11D-L3 and 28D-L1), mock and wild type plants, by dipping in chloroform for 15 s and evaporated to obtain dried wax (Mamrutha et al., 2010). The dried wax dissolved in hexane and injected to gas chromatograph–mass spectrometry (GC-MS) for the analysis of wax components (Coutinho et al., 2009). GC-MS analysis was carried out on a GC CLARUS 550 PerkinElmer system comprising a gas chromatograph interfaced to a mass spectrometer (GC-MS) instrument employing the following conditions: column Elite-1 fused silica capillary column (30 × 0.25 mm ID × 1EM df, composed of 100% dimethyl poly siloxane), operating in electron impact mode at 70 eV; helium (99.999%) was used as carrier gas at a constant flow of 1 ml/min and an injection volume of 0.5 EI was employed with split ratio of 10:1 injector temperature 250°C; ion-source temperature 280°C. The oven temperature was fixed from 110°C (isothermal for 2 min), with an increase of 10°C/min, to 200°C, then 5°C/min to 280°C, ending with a 9 min isothermal at 280°C. Mass spectra were taken at 70 eV; a scan interval of 0.5 s and fragments from 40 to 550 Da.

In this method, volatile compounds were separated from the sample matrix by passing inert gas helium through the matrix. The sample was placed in a sealed container, vial, and left at a constant temperature until the gas and liquid phase are in equilibrium. The target substances in the gas phase (headspace) were collected by gas tight syringe. This is injected into the GC/MS. Calibration curve were made by dissolving the target chemicals in purified water, and then treated in the same manner. The target, volatile compounds are desorbed from the aqueous phase to the gas phase (purged) and are then separated from the stream of gas (trapped) by adsorbent filters. The adsorbent material is then heated in a stream of GC carrier gas (usually pure helium). This releases the trapped substances into the carrier gas, the target analytes were introduced to GC, and analyzed. Typical trapping (adsorbent) material was silica gel and other GC column packing materials, or combinations of such materials.

### Physiological and Biochemical Analysis of Transgenic Groundnut Plants Exposed to Drought Stress

To analyze the degree of endurance to water stress at the whole-plant level, *AhKCS1* transgenic groundnut plants (11D-L3 and 28D-L1), mock (empty vector), wild type, and cultivar K-9 were grown in pots containing red soil and farm yard manure in 2:1 ratio. One-month-old plants were subjected to water stress by withholding water to maintain 25% SML as measured gravimetrically, for 10 days and leaf samples were collected for biochemical and physiological experimentations.

### Epicuticular Wax Quantification

Waxes exterior to the leaf surface were separated and quantified by colorimetric assay (Mamrutha et al., 2010). According to this method, waxes react with acidic-potassium dichromate (K_2_Cr_2_O_7_) to give a colored complex, where carnauba wax (Sigma, United States) is used as a standard for wax quantification (Samdur et al., 2003). The wax content was measured as μg/gm fresh weight.

### Lipid Peroxidation

The lipid peroxidation was quantified by determining the 2-thiobarbituric acid reactive substances mainly Malondialdehyde (MDA). MDA content was determined according to the method of Hodges et al. (1999).

### Chlorophyll Leaching Assay

For chlorophyll leaching assay, mature leaves were washed with tap water and kept in tubes containing 20 mL of ethanol (80%, v/v) at room temperature (gently agitating in the dark). The amount of chlorophyll extracted into the solution was estimated every 30 min upto 5 h after recording the absorbance at wavelengths 663 and 645 nm using a spectrophotometer (Shimadzu UV-1800 Japan) (Hiscox and Israelstam, 1979).

### Rate of Water Loss

The plants were kept in constant dark for 10 h to allow plant transpiration rates to stabilize, then the leaves (1 gm) were detached and initial weight at different time intervals was recorded. Measurements were made at 28°C and 50% RH in a dark room. The detached leaf samples were dried for 24 h at 80°C and final dry weight was recorded. The rate of water loss was the difference between initial and final weights. The results are average of three replicates.

### Moisture Retention Capacity

Leaves harvested early in the morning and fresh weight was recorded immediately at hourly intervals up to 5 h (Mamrutha et al., 2010). The experiments were conducted at constant temperature (30 ± 0.5°C) and RH (55–60%) under a light intensity of 500–550 mmol m^-2^ s^-1^. At end of the experiment, leaves were dried to a constant weight in a hot air oven at 80°C for 24 h.

The moisture retention capacity (MRC) was calculated using the formula: MRC (%/) = [(FW1 - DW) / (FW0 - DW)] × 100 where, FW0 is the fresh weight (g) immediately after harvest, FW1 is the weight (g) at a particular hour after harvest and DW is the oven dry weight (g).

### Cell Membrane Stability

The membrane damage was assessed by electrolyte leakage assay as defined by Leopold et al. (1981). The percent leakage was measured using the formula, EC (%) = Initial EC/Final EC × 100.

### Relative Water Content

The relative water content (RWC) content was calculated according to Barrs and Weatherly (1968). The RWC was calculated using the formula RWC = Fresh weight (FW) - Dry weight (DW)/Turgid weight (TW) - Dry weight (DW).

### Free Proline Content

Free proline was measured from the standard curve and calculated on μmol/g sample from the protocol as reported by Bates et al. (1973).

### Statistical Analysis

All the experiments were conducted in three biological replicates. Data presented are mean values and standard deviation (±SD). One-way ANOVA was done using *Post hoc* multiple comparison from the Duncan’s test at a significance level of *p* = 0.05.

## Results

### PCR Amplification and Sequence Analysis of *AhKCS1* Gene

PCR amplification of *AhKCS1* gene resulted in an amplicon of ∼1.4 kb (Supplementary Figure 6). Sequencing and sequence analysis of *AhKCS1* revealed that the gene was 1491 bp. The sequence was submitted to genbank with Accession KM886246 (Supplementary Figures 1–3). Phylogenetic analysis affirmed that the *AhKCS1* gene corresponds to the condensing enzyme family of KCS (Supplementary Figure 4).

### Agrobacterium Mediated Transformation and Production of Groundnut Transgenic Plants

The binary vector (*pC2301:35S: NptII: AhKCS1: GUS*) construct (Supplementary Figure 5A) was confirmed by restriction analysis, mobilized into *Agrobacterium* strain EHA105. The gene *AhKCS1* was introduced into groundnut cultivar K-6 seedlings by tissue culture-independent *in planta* transformation procedure (Rohini and Rao, 2001). The transformants of T1 plants were screened for kanamycin resistance. Putative transformants exhibited resistance against kanamycin (200 mg/L) due to presence of integrated transgene construct and showed normal, while wild type did not grow (Supplementary Figure 5B). Only resistant healthy plants were chosen and advanced for next transgenic generation.

### Screening of the *AhKCS1* Transgenic Plants by Reverse Transcription – PCR

PCR was employed to screen the presence of transgene in genomic DNA from the wild-type, mock, and putative *AhKCS1* transformants using *NptII* (selectable marker) primers, GUS gene specific primers and with *AhKCS1* gene specific primers. The transformants revealed the amplifications of 1491 bp fragment with *AhKCS1* specific primers (Supplementary Figures 6A,B) and 500 bp fragments with both *NptII* and GUS primers, whereas wild type showed no amplification (Supplementary Figures 6C,D).

### SYBR Green-Based Real Time qRT-PCR Analysis

Among the *NptII*, GUS and *KCS1* gene PCR-positive transgenic groundnut plants, best performing lines (11D-L3 and 28D-L1) were analyzed for *AhKCS1* gene expression analysis using SYBR Green qRT-PCR. They showed enhanced transgene expression, as evidenced by qRT-PCR results (Supplementary Figure 8). The qRT-PCR results inferred that the transcript levels of *AhKCS1* gene was increased by three to fourfolds in transgenic groundnut plants, compared to mock and wild type plants.

### Scanning Electron Microscopy-Based Surface Analysis of Transgenic Plant Leaves

**FIGURE 1:**
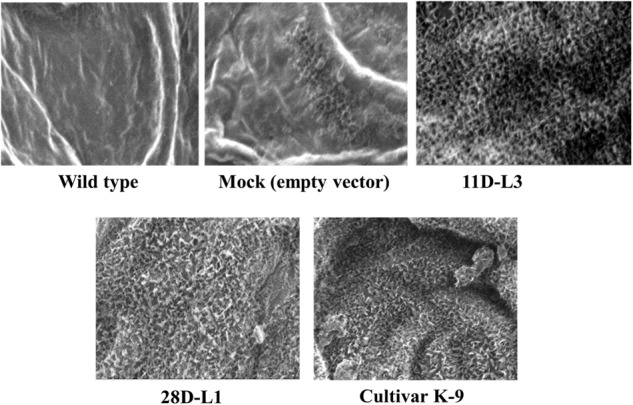
Analysis of wax crystal morphology of groundnut transgenic, mock, and wild type plants using Environmental Scanning Electron Microscope (QUANTA 250 ESEM). ESEM image showing the variation in epicuticular wax deposition on the leaf surfaces of *AhKCSl* groundnut transgenic lines 11D-L3, 28D-L1, mock (empty vector), Cultivar K-9, and wild type under drought stress with a magnification of 2000× at 40 μm bar scale.

ESEM analysis confirmed the difference in leaf epicuticular wax crystal morphology among *AhKCS1* transgenic lines (11D-L3 and 28D-L1), mock, wild type, and cultivar K-9 groundnut plants. Over expression of *AhKCS1* was associated with alterations in the epicuticular wax crystals deposition both on the adaxial and abaxial surfaces of the leaf. Compared to adaxial surface, there were less dense wax crystals deposited on the abaxial surface of groundnut transgenic leaves. The surface of the transgenic leaves (11D-L3 and 28D-L1) exhibited dense wax crystals accumulation, onpar with cultivar K-9, whereas the mock and wild type plants have sparse wax accumulation (Figure 1).

### GC-MS Analysis of Epicuticular Wax Components

**FIGURE 2:**
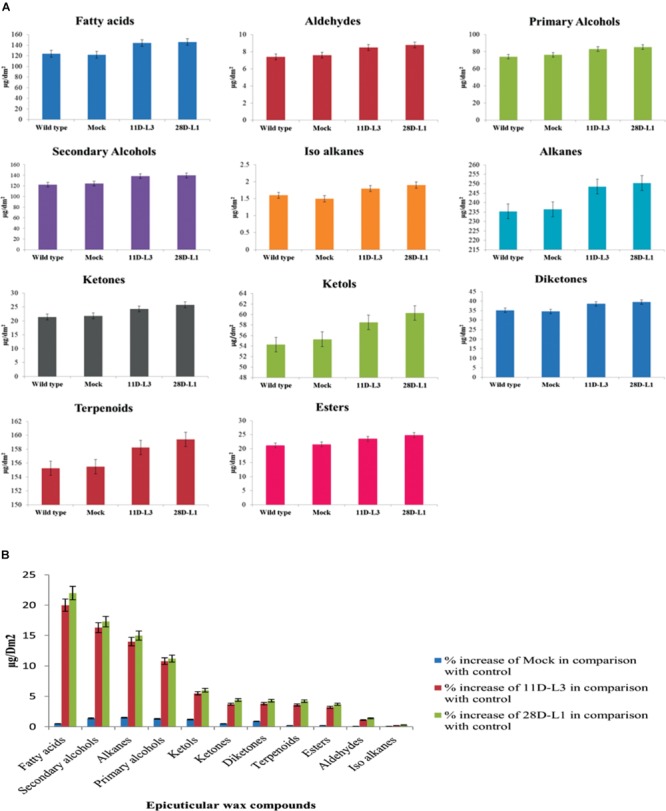
Analyses of leaf wax components in *AhKCSl* transgenic, mock (empty vector) and wild-type groundnut cultivar K6 by GC-MS. **(A)** Leaf wax components in *AhKCSl* transgenic lines 11D-L3 and 28D-L1, mock (empty vector) and wild type, groundnut cultivar K6 showing higher alcohols, alkanes, ketols, ketones, and esters in leaves. **(B)** Percent increase of wax compounds in two *AhKCSl* transgenic groundnut leaf samples 11D-L3, 28D-L1, and mock when compared to wild type. The results show average of three replicates and error bars indicate mean ± SE.

Wax components of the leaf samples of transgenic groundnut lines (11D-L3 and 28D-L1), mock and wild type were analyzed by GC-MS. There were significant differences in wax components across the transgenic groundnut plants, mock, and wild type (Figure 2A). Epicuticular wax components, such as Fatty acids increased by 22%, Secondary alcohols 17.3%, Alkanes 15%, Primary alcohols 11.2%, Ketols 6%, Ketones 4.4%, Diketones 4.3%, Terpenoids 4.2%, Esters 3.7%, Aldehydes 1.4%, and Iso alkanes 0.3% form transgenic groundnut plants compared to mock and wild type plants (Figure 2B).

### Drought Stress Analysis

**FIGURE 3:**
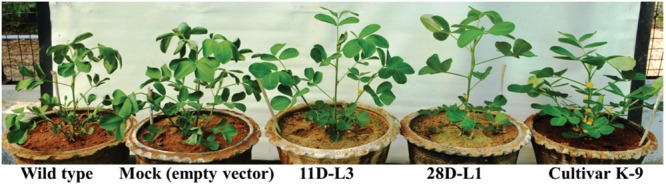
Drought stress analysis *of AhKCSl* transgenic groundnut lines, mock, Cultivar K-9, and wild type plants. *AhKCSl* transgenic groundnut lines showing stay green nature, along with cultivar K-9 and visible wilting symptoms in mock and wild type plants 10 days after drought stress. WT-wild type, M-mock, 11D-L3, and 28D-L1 – *AhKCSl* transgenic groundnut lines.

*AhKCS1* transgenic plants and cultivar K-9 showed much delayed leaf wilting symptoms compared to mock and wild type plants, 10 days after drought stress (Figure 3).

### Effect of Drought Stress on Epicuticular Wax Content

**FIGURE 4:**
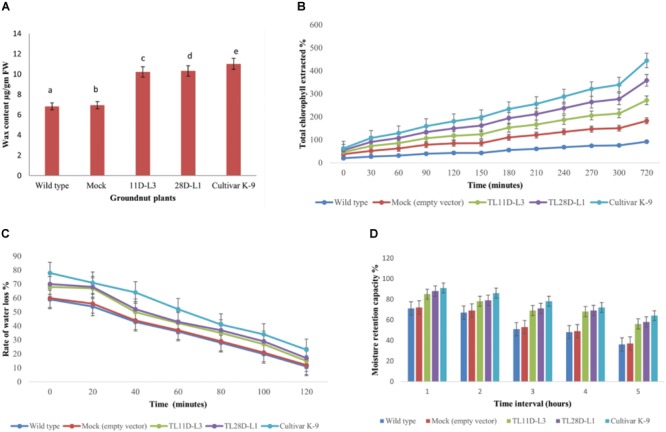
*AhKCSl* transgenic groundnut lines 11D-L3, 28D-L1, mock, Cultivar K-9, and wild-type one-month-old plants subjected to 10 days of drought stress and the leaves were collected to estimate different bio-chemical parameters. **(A)** Estimation of Epicuticular wax content showing increased accumulation of EWC in transgenic lines, onpar with Cultivar K-9. **(B)** Chlorophyll leaching assay, at different time points, showing the % of chlorophyll extracted was very less in transgenic lines, a little less than that of Cultivar K-9. **(C)** Rate of water loss was low in transgenic lines, Cultivar K-9 compared to mock and wild type. **(D)** Estimation of Moisture Retention Capacity revealed that transgenic lines retrained more water in the harvested leaves, along with Cultivar K-9. Values shown are the mean of three replicates and ±SE of three replicates and letters shown above the bars are significantly different at *P* < 0.05.

From the existent study a significant variation was noticed in EWC ranged from 6.8 to 11.01 μg/gm FW in wild type, cultivar K-9, mock, and transgenic groundnut plants imposed to drought stress for 10 days (Figure 4A). Transgenic groundnut lines (11D-L3 and 28D-L1) demonstrated significantly higher EWC (10.21 and 10.32 μg/gm FW), slightly lower than cultivar K-9 (11.01 μg/gm FW) in comparison with mock and wild type plants (6.93 and 6.82 μg/gm FW).

### Chlorophyll Leaching Assay

The chlorophyll leaching assay was done from *AhKCS1* transgenic groundnut lines (11D-L3 and 28D-L1), mock, wild type, and cultivar K-9 plants exposed to drought stress for 10 days. The chlorophyll leaching rate was much lower in *AhKCS1* transgenic (11D-L3 and 28D-L1) and cultivar K-9 leaves, as compared to mock and wild type (Figure 4B).

### Rate of Water Loss

Rate of water loss was calculated using the leaves of *AhKCS1* transgenic groundnut lines 11D-L3 and 28D-L1, mock, cultivar K-9, and wild type indicated that, the water loss was high in the leaves of mock and wild type compared to leaves of cultivar K-9 and *AhKCS1* transgenic groundnut lines 11D-L3 and 28D-L1 (Figure 4C).

### Moisture Retention Capacity

The MRC was examined in *AhKCS1* transgenic groundnut lines, mock, wild type, and cultivar K-9 groundnut plants. The results revealed that *AhKCS1* transgenic groundnut lines 11D-L3 and 28D-L1 maintained high leaf moisture content (52–56%) significantly higher, but slightly less than that of cultivar K-9 (58%) compared to mock and wild type (35 and 37%) during drought stress (Figure 4D).

### Lipid Peroxidation

**FIGURE 5:**
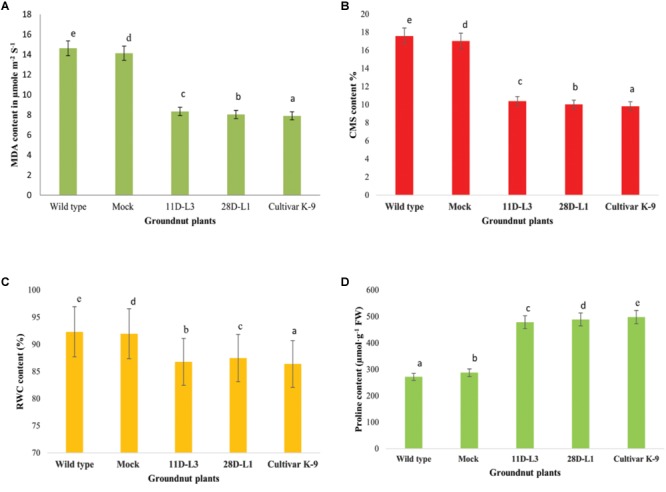
Leaf material was collected from one month old transgenic groundnut lines 11D-L3, 28D-L1, mock, Cultivar K-9, and wild-type plants subjected to 10 days of drought stress to estimate different bio-chemical parameters. **(A)** Malondialdehyde (MDA) content was low in transgenic lines and Cultivar K-9. **(B)** Cell membrane stability (CMS) in transgenic lines and Cultivar K-9 was high with low electrolyte leakage. **(C)** Relative water content % (RWC) was less in transgenic lines and Cultivar K-9. **(D)** Proline content was high in transgenic lines and Cultivar K-9. Values shown are the mean of three replicates and ±SE of three replicates and letters shown above the bars are significantly different at *P*<0.05.

MDA accumulation is used as indicator of membrane damage. Leaf MDA content was analyzed in *AhKCS1* transgenic groundnut lines (11D-L3 and 28D-L1), mock, wild type, and cultivar K-9 to assess the membrane integrity under water deficit treatment. The MDA content in the leaves of *AhKCS1* transgenic groundnut lines, mock, wild type, and cultivar K-9 increased after 10 days of drought stress, but the level was much lower in transgenic lines and cultivar K-9 than in mock and wild type plants. The MDA content was 14.62 μmol m^-2^ S^-1^, in wild type and 14.13 μmol m^-2^ S^-1^ in mock plants and 8.33 and 8.03 μmol m^-2^ S^-1^ in transgenic groundnut lines, 11D-L3 and 28D-L1, 7.9 μmol m^-2^ S^-1^ in cultivar K-9, respectively (Figure 5A).

### Cell Membrane Stability

The cell membrane stability (CMS) was estimated as electrolyte leakage in *AhKCS1* transgenic groundnut lines (11D-L3 and 28D-L1), mock, wild type, and cultivar K-9 plants, 10 days after drought stress. The electrolyte leakage in *AhKCS1* transgenic groundnut (11D-L3 and 28D-L1) plants was (10.39 and 10.02%), and cultivar K-9 was (9.83%) significantly lower, compared to mock and wild-type plants (17.05 and 17.59%) during drought stress (Figure 5B).

### Relative Water Content

In general, RWC decreased in *AhKCS1* transgenic groundnut (11D-L3 and 28D-L1) lines, mock, wild type, and cultivar K-9 plants under drought stress. However, in *AhKCS1* transgenic groundnut (11D-L3 and 28D-L1) plants RWC was minimally reduced by 13.82 and 12.65%, which was slightly less than that of cultivar K-9 by 11.95% compared to watered wild type control and mock which were 8.45% and 9.32%, respectively (Figure 5C).

### Free Proline Content

Free proline content of the transgenic groundnut lines (11D-L3 and 28D-L1) was (479 and 489 μmol/g^-1^ FW), higher than that of mock and wild-type (288 and 272 μmol/g^-1^ FW) plants but less than that of cultivar K-9 (498 μmol/g^-1^ FW) during drought stress (Figure 5D).

## Discussion

Cuticular waxes exterior to the organs of the plant play a critical role in sustaining to the harsh environmental conditions such as drought (Jenks and Ashworth, 1999; Goodwin and Jenks, 2005). Drought stress triggers the increased deposition of waxes in many plants (Bondada et al., 1996; Samdur et al., 2003; Cameron et al., 2006). The cuticular waxes ensure plants to limit transpiration, enhance water conservation, and keepup profound growth under water-limiting conditions (Schonherr, 1982; Sanchez et al., 2001; Goodwin and Jenks, 2005).

It is rather true that, plants when exposed to stress, adopt several approaches to sustain and one such approach would be the epicuticular wax accumulation, to minimize water loss from the leaf exterior. In the current study, qRT-PCR analysis showed elevated levels of *KCS1* transgene expression in two transgenic groundnut plants during drought-stress, compared to mock and wild type plants. Two transgenic groundnut plants showed over four folds increase in total wax content confirm that *AhKCS1* expression enhanced wax biosynthesis and secretion pathways under drought stress, as described earlier in *Arabidopsis* (Todd et al., 1999), *Brassica napus* (Chiron et al., 2015), apple (Hao et al., 2017). In this context, higher expression of the *AhKCS1* gene seems a better survival strategy under stress condition in transgenic groundnut plants.

In the present study, the leaf surface of transgenic groundnut lines 11D-L3, 28D-L1, and cultivar K-9 revealed the presence of dense wax crystals apparently under high magnification of 2,000X compared to mock and wild type plants. This clearly signifies that overexpression of *AhKCS1* resulted in the deposition of epicuticular waxes in transgenic groundnut plants, similar to cultivar K-9 to impart drought tolerance. Further the wax composition between transgenics, mock and wild type plants was analyzed by GC-MS. Transgenic groundnut lines 11D-L3 and 28D-L1 displayed an increase of 22% in the fatty acids, 17.3% in secondary alcohols, 15% in alkanes, 11.2% in primary alcohols, 6% in ketols, 4.4% in ketones, 3.7% in wax esters, 1.4% in aldehyde content when compared to mock and wild type plants. Our results were in concurrence with previous data reported from crop plants like soya bean, exhibiting significant increase in alkanes (28%), primary alcohols (11%), and triterpenoids (39%) (Kim et al., 2007) and potato showing enhancement in 2-methylalkanes and 3-methylalkanes (3.1–4.6 μgcm^-2^), primary alcohols (0.3–0.7 μgcm^-2^), Fatty acids (0.3–0.6 μgcm^-2^), wax esters (0.1–0.4 μgcm^-2^) (Szafranek et al., 2005).

In our current study, we observed the presence of secondary alcohols, alkanes, and esters in higher amounts and aldehydes in lower amounts in transgenic groundnut plants. Increase in the content of secondary alcohols, alkanes, and lower levels in the quantity of alcohols and ester compounds forms uniform barricade opposing water loss, because of their lipophilic nature with longer carbon chain tends to bridge the gaps present in the porous wax layer (Niederl et al., 1998; Shephered and Griffiths, 2006). Our results were in concomitant with the previous studies on cotton, wheat, oats, and peanut (Bengtson et al., 1978; Rao et al., 1981; Xu et al., 1995; Bondada et al., 1996). A disproportionate build-up of secondary alcohols and alkanes, in contrast with the lesser amount in ester and aldehyde levels in transgenic groundnut lines, demands a preferential influx by the decarbonylation pathway, rather than the acyl reduction pathway, as previously reported in *Arabidopsis WIN1*/*SHN1* gene (Aharoni et al., 2004; Broun et al., 2004).

Furthermore, *AhKCS1* transgenic groundnut lines have high chlorophyll retention capacity under drought stress, as that of cultivar K-9 compared to mock and wild type, which was previously reported by Kosma et al. (2009), Amara et al. (2013), and Mackova et al. (2013). Rate of water loss was relatively low in transgenic groundnut plants and cultivar K-9, than in mock and wild type plants under drought stress. Our results were in concomitant with the previous investigations in sorghum cultivars (Chatterton et al., 1975; Premachandra et al., 1994; Jenks et al., 1994). The leaf moisture content determines the leaf metabolic activity and hence MRC is very crucial in plants exposed to water limited conditions. The *AhKCS1* transgenic groundnut plants maintained significantly higher MRC, equal to that of cultivar K-9 than that of mock and wild type plants as supported by previous findings suggesting the regulation of cuticular transpiration by Mamrutha et al. (2010) in mulberry (Bengtson et al., 1978; O’Toole et al., 1979; Jefferson et al., 1989; Bourdenx et al., 2011; Zhu et al., 2014; Anbazhagan et al., 2015).

Abiotic stress accords negative effects on membrane integrity. Electrolyte leakage and malondialdehyde accumulation are the indicators of membrane damage. The transgenic groundnut plants exhibited lower MDA content, as reported from tobacco (Wang et al., 2013) and poplar (Chu et al., 2016), showed high CMS, as previously reported by Franca et al. (2000), contained high RWC, as reported in maize (Amara et al., 2013) and alfalfa (Wang Z. et al., 2014), high free proline content as previously reported from alfalfa (Wang Z. et al., 2014), peanut (Bhauso et al., 2014; Sarkar et al., 2014) similar to that of cultivar K-9, upon comparison with mock and wild type plants.

## Conclusion

In summary, the transgenic groundnut plants showed improved traits like enhanced epicuticular wax accumulation, reduction in cuticular transpiration, lower membrane damage, high cell membrane stability, and high free proline content due to the overexpression of *AhKCS1* gene in sensitive groundnut cultivar K-6 exhibiting enhanced drought tolerance. Hence, our study demonstrates that *KCS1* would be the potential gene to improve stress tolerance in drought susceptible groundnut cultivars.

## Ethics Statement

All the experiments were approved by RCGM, Government of India No./BT/BS.17/686/2016-PID.

## Author Contributions

CS conceived and designed the experiments. UL, BV, KK, and AN performed the experiments. UL and AMAJ carried out the sequence analysis and annotation. CS, UL, and AMAJ wrote the manuscript. GLR, VA, and MP provided the suggestions and inputs for experimentation.

## Conflict of Interest Statement

The authors declare that the research was conducted in the absence of any commercial or financial relationships that could be construed as a potential conflict of interest.
