# Supplementary material for: Overexpression of ß-Ketoacyl Co-A Synthase1 Gene Improves Tolerance of Drought Susceptible Groundnut (Arachis hypogaea L.) Cultivar K-6 by Increased Leaf Epicuticular Wax Accumulation
Source: Front Plant Sci. 2019 Jan 11;9:1869. doi: 10.3389/fpls.2018.01869 (PMC6336926; doi:10.3389/fpls.2018.01869)
Supplement: Supplementary file 1 [file Image_1.pdf]

```
atgcctcccatgttgccggatttctccaactccgtgaagctcaagtatgtcaagcttggatacc
aataccttgttaaccacattatcacactcaccctcgttccaatcatgctgggagtctccattga
gattctacgcttaggcccccaagagatccttaatctctggaattccctgcacttcaacctcgtt
cagatcctctgctccgctttcctcatcatcttcgttgccaccgtctacttcatgtcaaagccac
gtacaatttacctcgttgactatgcttgcttcaagccaccggtaacatgccgggtccccttcgc
caccttcatggagcactcaaggctcatcctcaagaacaaccccaagagtgtggagttccagatg
aggatccttgagcgctccggcctcggcgaagagacctgtcttcctcctgccattcactacatcc
ctcccaagcctaccatggaggccgctcgcggcgaggccgagcttgtcatcttctcagccatgga
ctctttgtttaagaaaaccggcctcaagcctaaggatatcgacattctcatagtgaattgcagt
ctcttctctccaactccttccttgtccgccatggttatcaacaagtacaagctcaggagcaaca
tcaagagcttcaacctctcggggatgggttgcagtgcgggtctcatctccatagacctagcacg
cgatcttcttcaggttcatcccaattccaacgccgtcgttgtcagcactgagattatcacgcct
aactactaccaaggcaacgagagagccatgcttcttccgaactgcttgttcaggatgggcggcg
ccgccatcctcttgtcgaaccggagatcggaacggagaagagccaagtacagattggtccacgt
ggttagaactcacaagggtgccgatgacaaagcctaccgttgtgtgttcgaggaagaagacaaa
gaaggaaaggttgggatttcgctgtccaaagacctcatggccattgcaggggaagctttgaagt
cgaacatcacaaccatgggtccgcttgttcttccggcatcggagcagcttctcttccttctgac
actgattgggaggaaaatcttcaaccctaagtggaagccatacatccctgacttcaagcaagct
ttcgagcacttctgcatccacgcgggtggacgcgctgtgatcgacgagttgcagaagaatcttc
aactgtcgacggagcacgtggaagcttccagaatgacccttcacaggttcggcaacacttcgtc
ttcttctctgtggtatgaactgaactacattgaatcgaaagggaggatgaagaaaggggatagg
gtgtggcagattgctttcgggagtgggttcaaatgcaacagcgccgtgtggaagtgtaacaaga
ccattaagacccccattgatggaccttggactgattgcattgatcgttaccctgttcacattcc
tgagatcgttaagctctag
```

SUPPLEMENTARY FIGURE 1 Nucleotide sequence of *KCS1*gene showing 1491bp.
