# Supplementary material for: Overexpression of ß-Ketoacyl Co-A Synthase1 Gene Improves Tolerance of Drought Susceptible Groundnut (Arachis hypogaea L.) Cultivar K-6 by Increased Leaf Epicuticular Wax Accumulation
Source: Front Plant Sci. 2019 Jan 11;9:1869. doi: 10.3389/fpls.2018.01869 (PMC6336926; doi:10.3389/fpls.2018.01869)
Supplement: Supplementary file 2 [file Image_2.pdf]

M P P M L P D F S N S V K L K Y V K L G Y Q Y L V N H I I T L T
L V P I M L G V S I E I L R L G P Q E I L N L W N S L H F N L V
Q I L C S A F L I I F V A T V Y F M S K P R T I Y L V D Y A C F
K P P V T C R V P F A T F M E H S R L I L K N N P K S V E F Q M
R I L E R S G L G E E T C L P P A I H Y I P P K P T M E A A R G
E A E L V I F S A M D S L F K K T G L K P K D I D I L I V N C S
L F S P T P S L S A M V I N K Y K L R S N I K S F N L S G M G C
S A G L I S I D L A R D L L Q V H P N S N A V V V S T E I I T P
N Y Y Q G N E R A M L L P N C L F R M G G A A I L L S N R R S E
R R R A K Y R L V H V V R T H K G A D D K A Y R C V F E E E D K
E G K V G I S L S K D L M A I A G E A L K S N I T T M G P L V L
P A S E Q L L F L L T L I G R K I F N P K W K P Y I P D F K Q A
F E H F C I H A G G R A V I D E L Q K N L Q L S T E H V E A S R
M T L H R F G N T S S S S L W Y E L N Y I E S K G R M K K G D R
V W Q I A F G S G F K C N S A V W K C N K T I K T P I D G P W T
D C I D R Y P V H I P E I V K L **Stop**

SUPPLEMENTARY FIGURE 2 Aminoacid sequence of *AhKCS1* gene with Protein molecular of 56.03 KDa and Theoretical pI of 9.32.
