# Supplementary figures and images for: Overexpression of ß-Ketoacyl Co-A Synthase1 Gene Improves Tolerance of Drought Susceptible Groundnut (Arachis hypogaea L.) Cultivar K-6 by Increased Leaf Epicuticular Wax Accumulation
Source: Front Plant Sci. 2019 Jan 11;9:1869. doi: 10.3389/fpls.2018.01869 (PMC6336926; doi:10.3389/fpls.2018.01869)

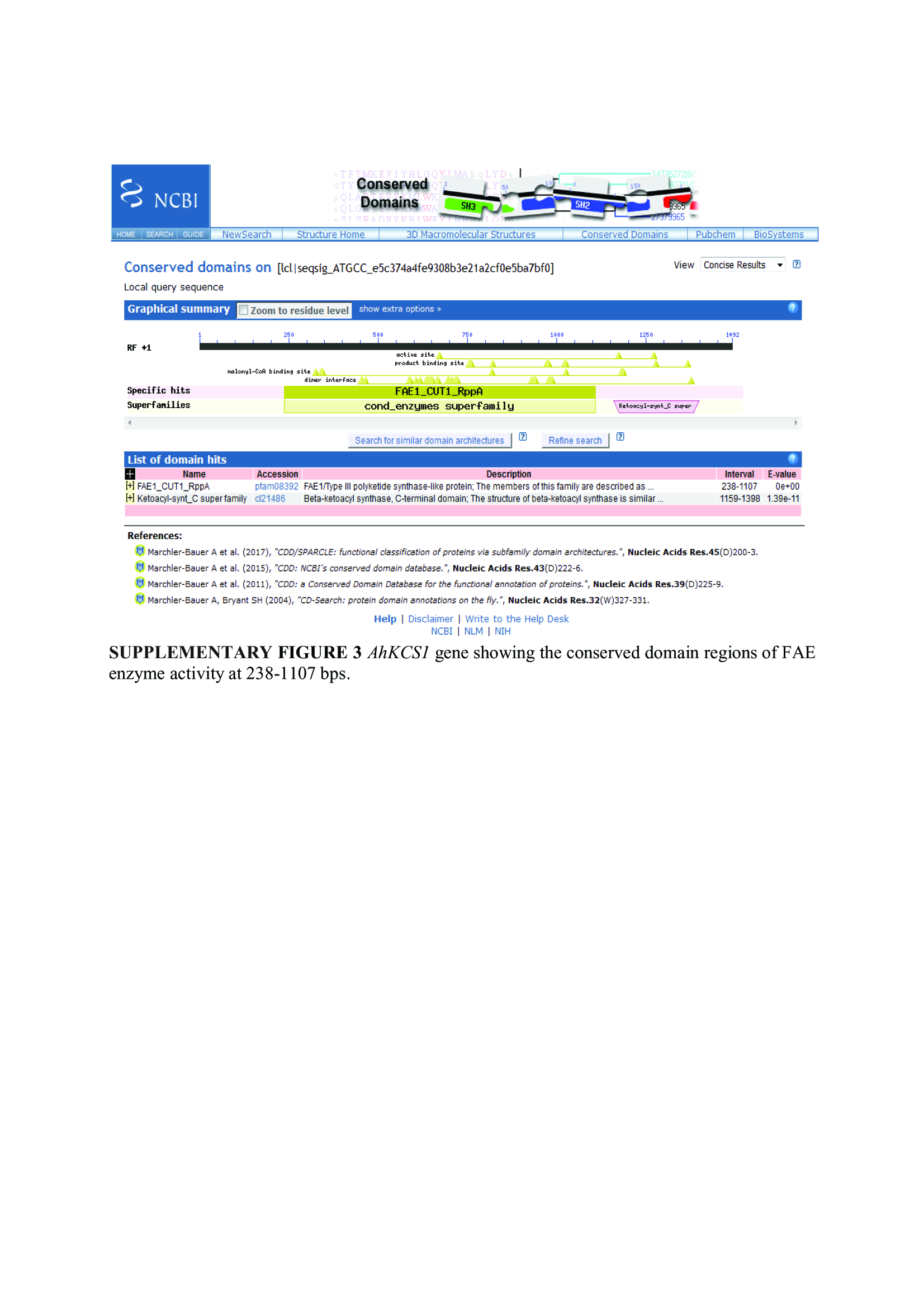

Supplement: Supplementary file 3 [file Image_3.tif]

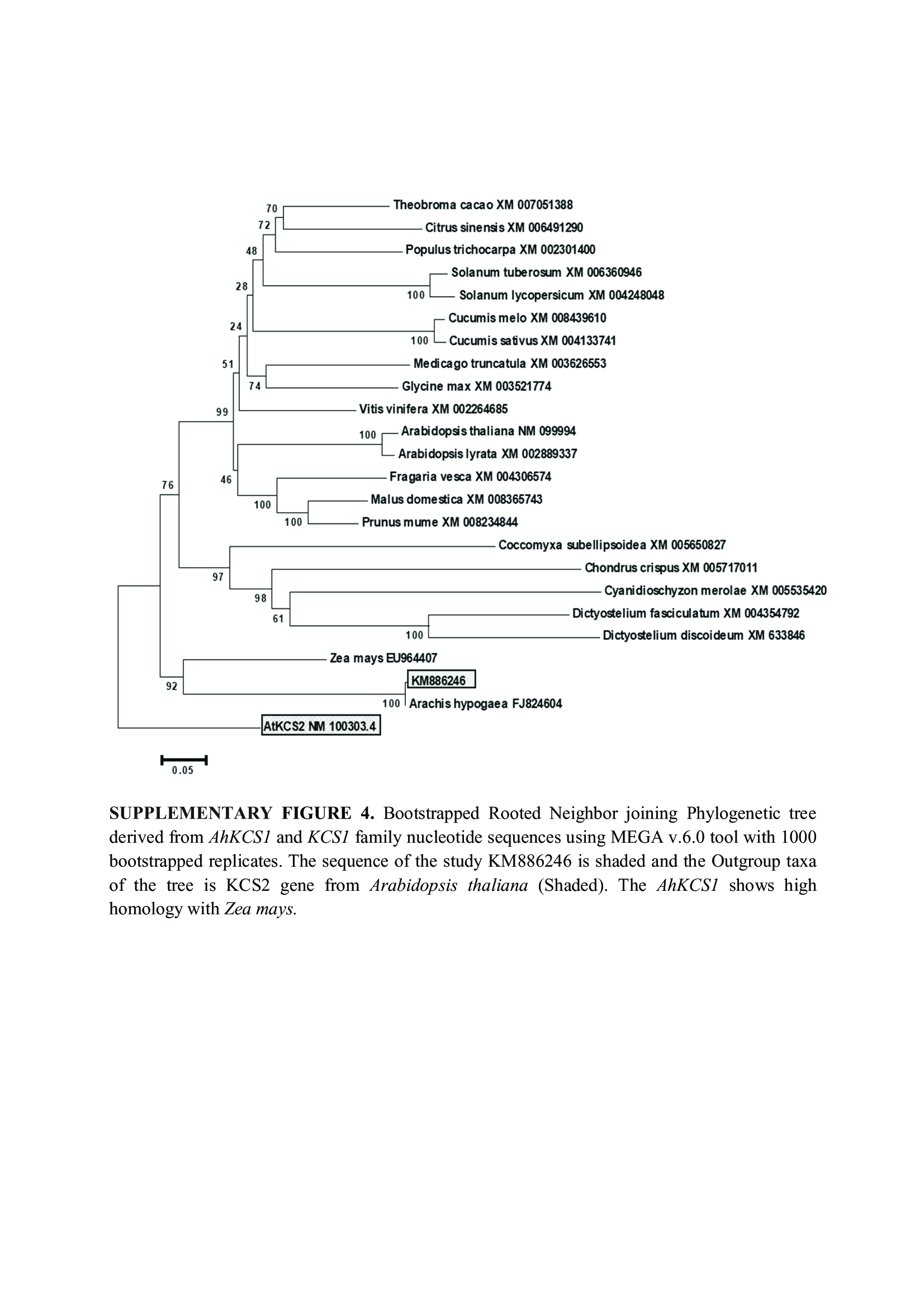

Supplement: Supplementary file 4 [file Image_4.tif]

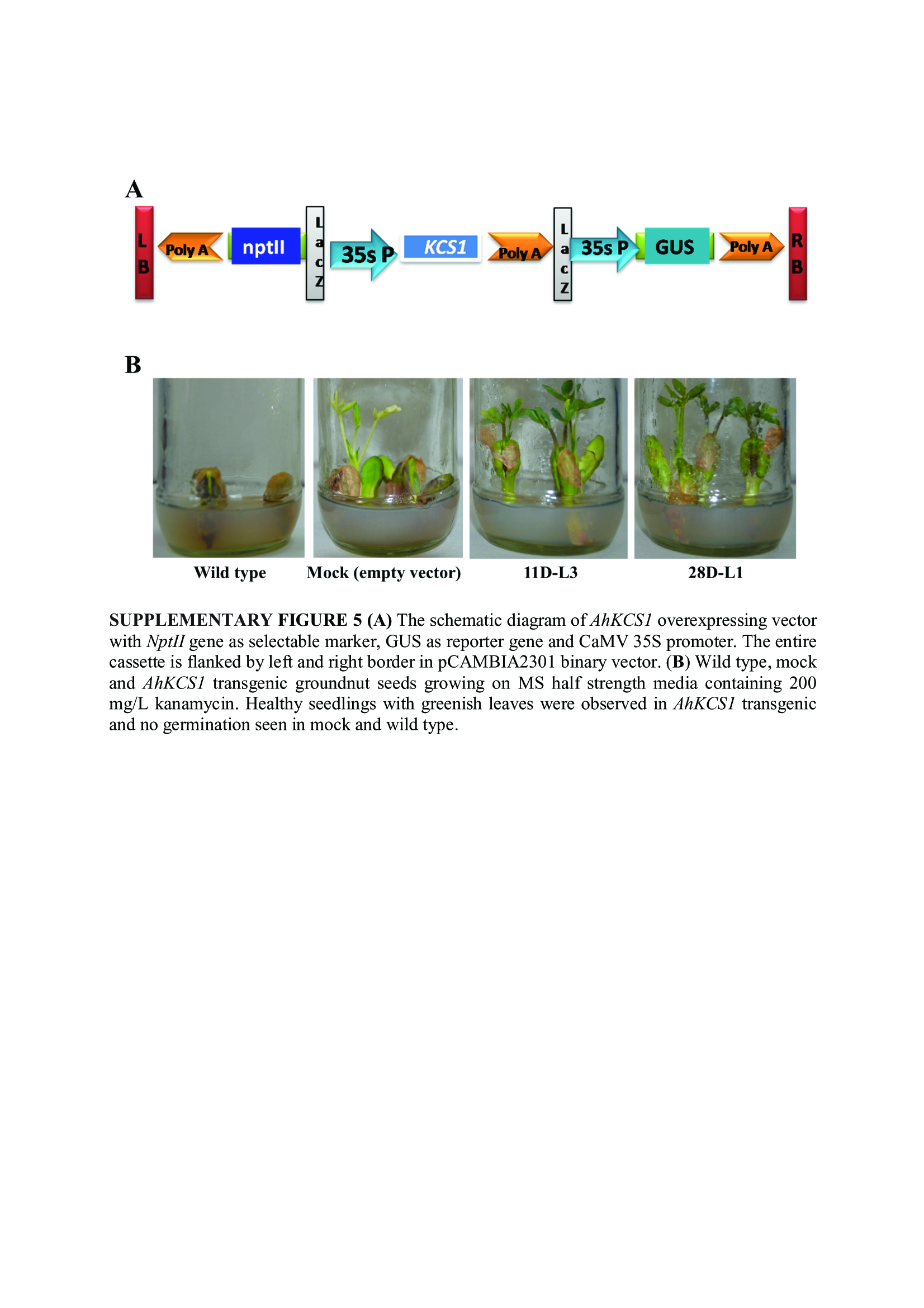

Supplement: Supplementary file 5 [file Image_5.tif]

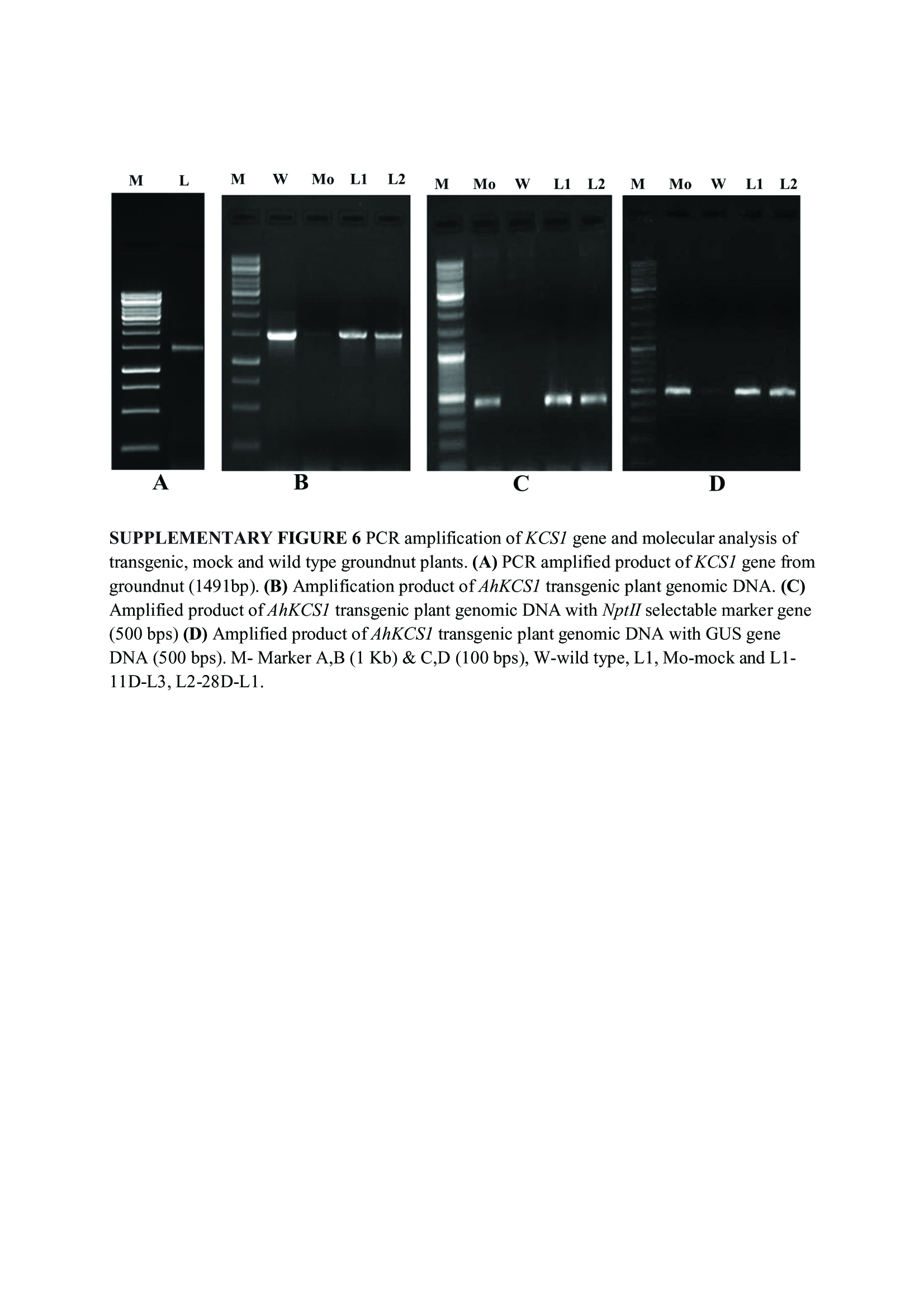

Supplement: Supplementary file 6 [file Image_6.tif]

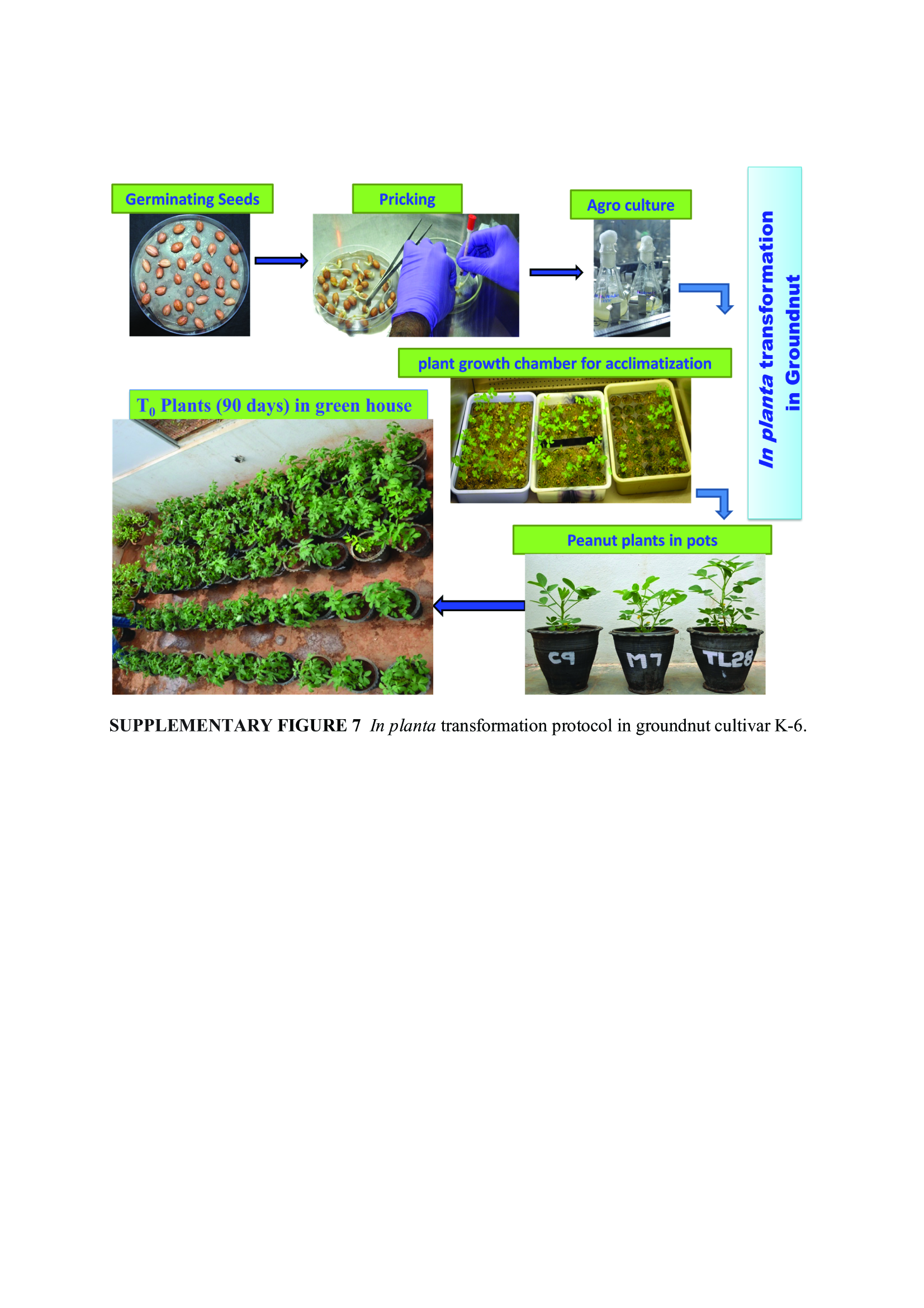

Supplement: Supplementary file 7 [file Image_7.tif]

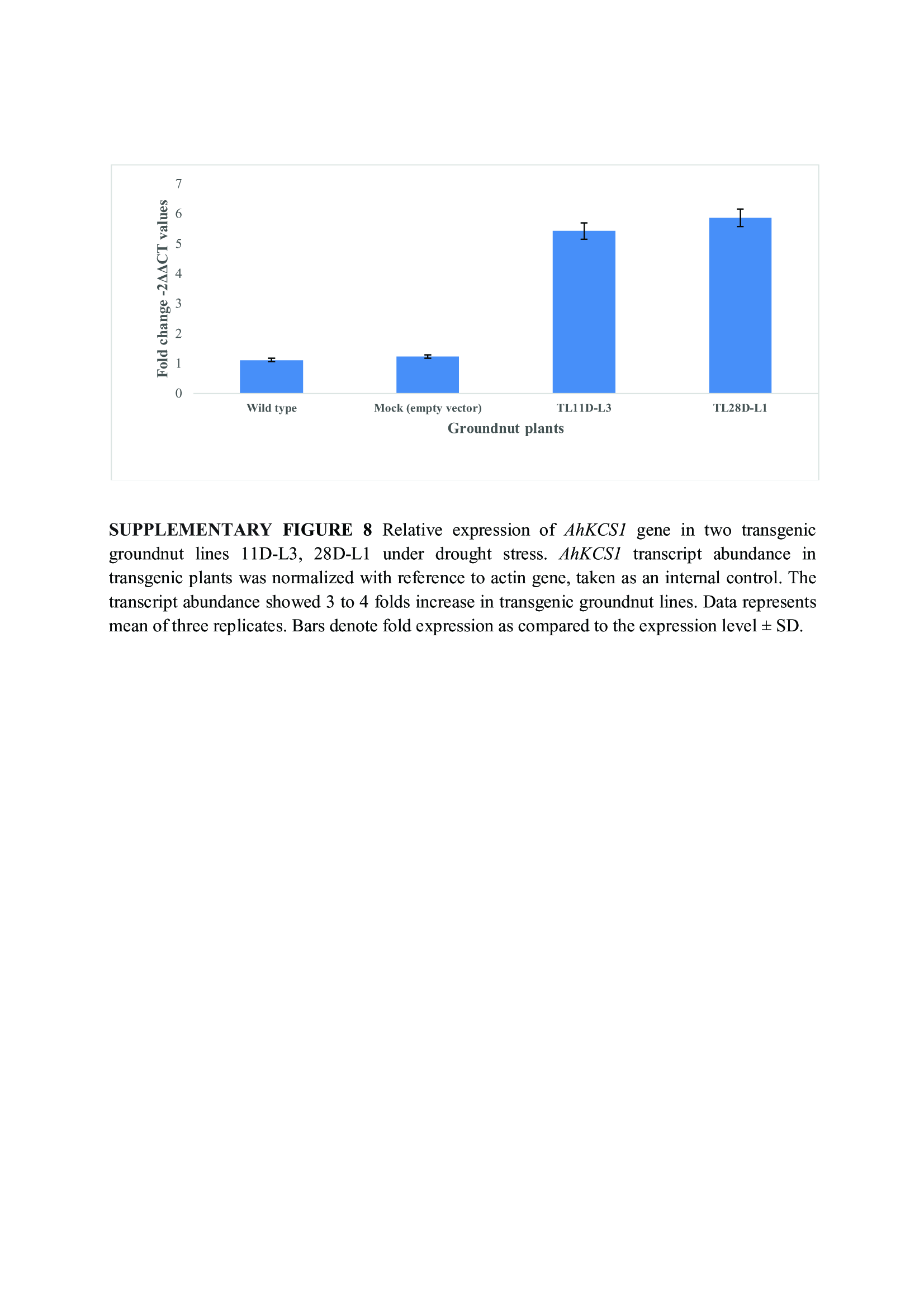

Supplement: Supplementary file 8 [file Image_8.tif]
